# COVID-19 Vaccination Knowledge, Attitudes, Perception, and Practices Among Frontline Healthcare Workers in Tunisia, 2024

**DOI:** 10.3390/vaccines14010074

**Published:** 2026-01-09

**Authors:** Fatma Ben Youssef, Aicha Hechaichi, Hajer Letaief, Sonia Dhaouadi, Amenallah Zouaiti, Khouloud Talmoudi, Sami Fitouri, Ahlem Fourati, Rim Mhadhbi, Asma Sahli, Ghaida Nahdi, Khouloud Nouira, Ihab Basha, Eva Bazant, Chelsey Griffin, Katie Palmer, Nissaf Bouafif ep Ben Alaya

**Affiliations:** 1 National Observatory of New and Emerging Diseases, Tunis 1002, Tunisia; aicha.hechaichi@gmail.com (A.H.); hejerletaief@gmail.com (H.L.); sonidhaouadi88@gmail.com (S.D.); zouaytiamen@gmail.com (A.Z.); talmoudi.khouloud@gmail.com (K.T.); fitourisami20@gmail.com (S.F.); fouratiahlem@gmail.com (A.F.); rim123mhadhbi@gmail.com (R.M.); asmasahli.mail@gmail.com (A.S.); naghmouchighaida@gmail.com (G.N.); khouloud.nouira.kn@gmail.com (K.N.); nissafba@yahoo.fr (N.B.e.B.A.); 2 Mediterranean and Black Sea Program for Intervention Epidemiology Training (MediPIET), European Center for Disease Prevention and Control (ECDC), 16973 Stockholm, Sweden; katie.palmer@ecdc.europa.eu; 3 Faculty of Medicine of Tunis, University Tunis El Manar, Tunis 1007, Tunisia; 4 The Task Force for Global Health, Decatur, GA 30030, USA; ebasha-consultant@taskforce.org (I.B.); ebazant-consultant@taskforce.org (E.B.); 5 Centers for Disease Control and Prevention (CDC), Atlanta, GA 30333, USA; qqp1@cdc.gov

**Keywords:** vaccines, knowledge, attitudes, perceptions, practices, vaccine hesitancy, primary healthcare

## Abstract

**Background/Objectives**: Healthcare workers (HCW) in primary care settings play a significant role in recommending vaccines to patients. We aimed to describe COVID-19 vaccination knowledge, attitudes, perception, and practices (KAPP) of HCWs in Tunisia and identify associated factors. **Methods**: We conducted a national cross-sectional survey (29 January to 3 February 2024) among HCWs in primary public healthcare centers using purposive sampling. Factors associated with good knowledge, positive attitude, and good practice, measured through Likert scales using face-to-face questionnaires, were identified using binary logistic regression. **Results**: We included 906 HCWs (mean age = 41.87 ± 8.89 years). In total, 37.75% (342/906) of HCWs had good knowledge and perception, 4.30% (39/906) had a positive attitude, and 24.9% (226/906) had good practices related to COVID-19 vaccination. Working in urban compared to rural areas was associated with good knowledge (aOR = 1.57, 95%CI = 1.12–2.21) and positive attitude (aOR = 4.94, 95%CI = 1.19–20.44) to COVID-19 vaccination. Physicians had better KAPP scores than other medical professionals. HCWs working in departments with high-risk patients were more likely to have good knowledge (aOR = 1.28, 95%CI = 1.00–1.72). Positive attitude was also associated with being male (aOR = 2.97, 95%CI = 1.75–5.07) and having at least one non-communicable disease (aOR = 1.92, 95%CI = 1.14–3.23). Being male (aOR = 1.97, 95%CI = 1.35–2.88) and having more years of professional experience (aOR = 1.81, 95%CI = 1.29–2.52) were associated with good practice. **Conclusions**: Just over a third of HCWs in primary healthcare clinics had good knowledge of COVID-19 vaccination, while positive attitudes and good practices were low. Targeted interventions, particularly for HCWs with less professional experience working in rural settings, are needed to increase good practices and improve COVID-19 vaccination coverage in Tunisia.

## 1. Introduction

Healthcare workers (HCWs) in primary public healthcare centers (PPHC) play a pivotal role in caring for individuals with COVID-19 [1]. Due to their interaction with infected individuals, HCWs face a heightened risk of contracting and transmitting infections like COVID-19, exposing themselves and vulnerable patients to potentially life-threatening complications [2]. Thus, international recommendations emphasize the importance of COVID-19 vaccination in HCWs [3,4]. Given the importance of protecting HCWs from severe COVID-19 forms and supporting continuity of healthcare services, some experts advocate for mandatory COVID-19 vaccinations for HCWs [5].

Attitudes toward vaccination, however, vary across and within different populations and were influenced by a range of factors during the COVID-19 pandemic [6,7]. Although willingness to receive a COVID-19 vaccine increased as the severity of the pandemic increased, it is important to reassess this in post-pandemic times [8]. Attitudes towards COVID-19 vaccines are multifaceted, involving psychological, social, and informational factors, which need to be continually assessed in HCWs [9].

As of October 2023, the National Observatory of New and Emerging Diseases (ONMNE) reported 1,156,613 COVID-19 cases and 29,494 deaths in Tunisia [10]. Five major outbreaks were reported between September 2020 and November 2025, with a Crude Mortality Rate (CMR) of 247.9 per 100,000 [11]. Between March and May 2020, 14.8% of COVID-19 national cases were among HCWs [12]. In Tunisia, COVID-19 vaccinations are widely recommended for HCWs [13]. The concomitant vaccination against COVID-19 and Influenza is also recommended [3]. However, vaccine hesitancy was reported in 51.9% of HCWs in a national study [14], which also highlighted that being female, working far from the capital city, and having concerns about the vaccines’ components were predictors of hesitancy among HCWs. Therefore, vaccine hesitancy among HCWs has been a significant barrier to the vaccination strategy [14,15]. However, several studies have proven the safety and effectiveness of COVID-19 vaccines, especially among immunocompromised patients [16,17]. In Tunisia, COVID-19 vaccines were also found to be effective in preventing SARS-CoV-2 infections and severe forms of COVID-19 [18].

HCWs in primary care settings play a significant role in recommending vaccines to patients [19]. Therefore, it is relevant to explore the factors that can influence their attitudes and perceptions regarding COVID-19 vaccination, as well as practices, including COVID-19 vaccine uptake and recommendations for appropriate patient groups. This is essential to orient and optimize strategies to improve vaccine uptake among this key population.

Given this context, ONMNE at the Tunisian Ministry of Health in collaboration with the US Centers for Disease Control and Prevention (CDC) (Partnership for Influenza Vaccine Introduction project), conducted a study that aimed to (i) describe the knowledge, perception, attitudes and practices of HCWs’ about COVID-19 vaccination (including vaccine updates and recommendations) and (ii) identify factors associated with good knowledge and perception of HCWs as well as positive attitude and good practice regarding COVID-19 vaccination.

## 2. Materials and Methods

The protocol was based on the US CDC protocol that was internally reviewed by the ONMNE team and adapted to the local context.

### 2.1. Survey Design: Period and Setting

We conducted a national cross-sectional survey among HCWs in Tunisia between 29 January and 3 February 2024.

### 2.2. Study Population

We included HCWs (medical physicians, nurses, nursing assistants, allied health professionals, medical/nursing students, and people with other clinical roles) working at PPHCs who agreed to participate in the study and were willing and able to provide informed consent.

#### 2.2.1. Inclusion Criteria

We included permanent HCWs (working for at least 6 months in the same place) in the selected PPHCs, providing direct clinical care to patients, and consenting to participate. Direct clinical care to patients refers to in-person, face-to-face contact between a healthcare provider and a patient, and may include treatment, screening, patient education, or any other aspect of healthcare of a patient [20].

#### 2.2.2. Exclusion Criteria

HCWs working in the selected PPHC who were not present on the day of the survey or refused to participate in the study were not included.

### 2.3. Sampling Method

#### 2.3.1. Sample Size Calculation

Given that our study involves multiple outcomes expressed as proportions and the population behavior is unknown, we applied Slovin’s formula to estimate the required sample size (n):

$$n\;=\;\frac{N}{{1\;+\;N(e)}^{2}}$$

where N = total population of target group, and e = desired precision (0.05).

We adjusted the sample size by a 2.5 design effect (DE) (assuming the worst-case scenario) using the following formula:

(1) $$\mathrm{DE}\;=\;1\;+\;m\alpha^{2}\backslash \mathrm{mu}$$

where m is the cluster size (we estimated the mean number of HCWs in the center to be 10), α is the Inter Correlation Coefficient (ICC = 0.05), and mu is the prevalence of the outcome (0.5).

As the expected size of each group’s total target population in the PPHCs was between 10,000 and 100,000, our target group included 1200 persons (with a minimum number of 900 individuals and an assumption of a 35% non-response rate).

#### 2.3.2. Sampling Process

We used a three-stage convenience sampling approach that aimed to include an average of 100 HCWs per governorate.

In the first stage, we randomly selected nine governorates out of a total of 24 (37.50%) from the three Great Regions of Tunisia (North, Center, South) as follows:

- From the North (3 out of 11): Tunis, Mannouba, Beja
- From the Center (4 out of 7): Kasserine, Kairouan, Monastir, Sidi Bouzid
- From the South (2 out of 6): Gafsa, Kebili

In the second stage, we selected the largest health districts (by number of HCWs) within the selected governorates. In each governorate, we randomly selected one health district from the list of those with at least 100 HCWs. If the number of eligible HCWs in the district was fewer than 100, we selected the next-largest health district (by number of HCWs) until we reached the required 100 HCWs per governorate.

In the third stage, PPHCs within each selected district were chosen using convenience criteria. All PPHCs in the selected health districts were included in the study, and all eligible HCWs at these centers were invited to participate. To ensure fieldwork feasibility, centers were prioritized according to proximity and workforce size. When the required number of HCWs could not be obtained from a given center, the nearest additional centers were subsequently included until the expected sample size was reached.

### 2.4. Data Collection and Management

Data were collected by trained interviewers, including medical physicians, Field Epidemiology Training Program (FETP) graduates, and family medicine residents. After obtaining written informed consent, the interviewers administered a face-to-face, standardized electronic questionnaire using the Computer-Assisted Personal Interviewing (CAPI) method via the KoboToolbox (Supplementary Materials).

The questionnaire included 41 questions covering (Supplementary Materials):

- HCW and PPHC characteristics, such as sociodemographic information and facility details.
- KAP scores, including questions on COVID-19 knowledge, perceptions, attitudes, and practices.

Primary COVID-19 vaccination was defined as completing a full primary series according to the type of COVID-19 vaccine: one dose of Janssen, two doses of AstraZeneca, Pfizer-BioNTech, Moderna, Sinopharm Sinovac, or Sputnik V.

A COVID-19 booster dose was defined as receiving one additional dose of a COVID-19 vaccine at least 3 months after the primary vaccination, regardless of vaccine type.

High-risk department units included Intensive Care Units (ICUs), emergency departments, and internal medicine departments.

High-risk groups for COVID-19 included individuals aged ≥ 65 years, HCWs, and persons with underlying chronic conditions, as defined in the National COVID-19 strategy [13].

Self-reported non-communicable diseases included obesity, diabetes, heart disease, lung disease, immunocompromised (weakened immune system due to a medical condition or from immunosuppressive treatments), hypertension, or other long-term conditions.

Types of centers included district hospitals, primary healthcare centers, and maternal and child health centers.

A threshold of 13 years was used to indicate the transition between early career and mid-career transition in HCWs [16,17].

The questions used to calculate the knowledge and perception score, attitude score, and practice score are shown in Table 2. The knowledge and perception score was assessed through four questions focusing on COVID-19 severity, COVID-19 vaccine safety, COVID-19 vaccine effectiveness in high-risk groups, and COVID-19 vaccines’ cross-protection with Influenza. The attitude score was obtained from five questions and assessed the willingness of HCWs to receive and recommend the COVID-19 vaccine in the future. The practice score was obtained from six questions and assessed COVID-19 vaccine uptake (primary series and booster doses) during the pandemic period and during the current season (2023–2024), and assessed whether HCWs recommend the COVID-19 vaccine to patients (primary series and booster doses).

For each question in the section, correct answers were defined according to national and international guidelines [13] and computed as 1, whereas a wrong answer was computed as 0. Not having an opinion or not answering was computed as 0. For Likert scale questions, we created a dichotomous variable: (i) agree (combining “agree” and “strongly agree”), (ii) disagree (combining “strongly disagree”, “disagree”, and “neutral”).

For each section, a total score was calculated by summing the number of correct answers for each question. This raw score was adjusted based on the total number of questions in each section to yield a percentage score.

KAPP scores were categorized as “good/positive”, “moderate”, and “low” using the percentage of correct answers. For each section, having more than 75% of good answers was classified as “good” or “positive”, having 50 to 74% of good answers as “moderate”, and having less than 50% of good answers was defined as “low”.

For univariable and multivariable analysis, the three main dichotomous outcomes were good knowledge, positive attitude, and good practice, and were defined as achieving ≥75% of correct/good answers in the respective sections.

We also investigated other variables, including sociodemographic factors (gender, age, years of experience as a HCW, and professional role) and work-related characteristics (urban/rural area of the PPHC and presence of non-communicable diseases in HCWs), which were self-reported.

The North region was taken as the reference region, as it includes the capital city, Tunis.

### 2.5. Data Analysis

Statistical analysis was performed using R Statistical Software version 4.1.2.

We described categorical variables using absolute numbers and percentages, and continuous variables using means or medians and interquartile ranges (IQRs).

In univariable analysis, we used Pearson’s chi-square tests for differences in percentages, Student’s *t*-test for normally distributed continuous variables, and Mann–Whitney U test for skewed distributions. Statistical significance was defined by a *p*-value of less than 0.05.

We presented the associations between good knowledge and perception, positive attitude, and good practice, and the explanatory variables, using crude odds ratios (ORs) and 95% confidence intervals (CIs).

Variables with a significant threshold of less than 0.2 were incorporated in the multivariable analysis. Multicollinearity was assessed using the Variance Inflation Factor (VIF) (values > 5 indicate collinearity), Tolerance, and Condition indices (values > 30 indicate multicollinearity).

Potential confounders associated with both exposure (explanatory factors) and outcomes were also included in the multivariate model as controlled variables. We used binary logistic regression in multivariable analysis to report adjusted OR (aOR) and 95%CIs.

The Hosmer–Lemeshow Goodness-of-Fit Test was used to assess the model fit. A non-significant result (*p* > 0.05) indicated good model fit.

### 2.6. Ethical Considerations

We obtained ethical approval for the study from the Ad Hoc Ethical Committee of the ONMNE (approval number 01_KAPP and date of approval 23 January 2024). Participation was voluntary, and all the participants were informed about the objectives and methods of the study before giving their written consent prior to the interview. Information on healthcare facilities was gathered during the initial survey process but was only stored in hard-copy files and not entered into the primary database. A unique identifier was assigned to each participant. We did not collect any personally identifiable information, such as names, family names, or dates of birth.

To ensure maximal response from participants, we obtained the Ministry of Health’s agreement and approval, as well as the Regional Healthcare Directorates’ approval, prior to data collection.

## 3. Results

### 3.1. Characteristics of the Study Population

Of the 1200 target sample, 906 HCWs (75.50%) were included.

The distribution of the included HCWs was homogeneous across governorates. The participants worked in 46 PPHCs distributed across 28 districts (Table 1).

The sex ratio (F/M) was 4.2. Participants’ ages ranged from 23 to 75 years, with a mean age of 41.87 ± 8.89 years. The median years of professional experience was 13.00 years (IQR 9.00–20.00 years). The main patient categories treated were children (85.65%, 776/906), adults older than 65 years (85.54%, 775/906), adults with non-communicable diseases (83.88%, 760/906), and pregnant women (80.68%, 731/906).

In total, 76.71% (695/906) of HCWs reported having made a clinical or laboratory-confirmed diagnosis of COVID-19 in a patient, and 59.49% (539/906) reported having treated a patient who developed a life-threatening complication (i.e., pneumonia or death) requiring hospitalization due to COVID-19.

### 3.2. HCWs’ Knowledge and Perception, Attitudes, and Practices Regarding COVID-19 Vaccines

The mean knowledge and perception score was 67.71% ± 24.64, ranging from 10% to 100%. One third (37.70%, 342/906) of HCWs had a good knowledge and perception score, while 31.60% (286/906) had a moderate score and 30.70% (278/906) had a low score.

Attitude score varied from 0 to 100% with a median score of 0% with IQ [0–42.86%]. Overall, 4.30% (39/906) had a positive attitude score, 14.68% (133/906) of HCWs had a moderate attitude score, and 81.02% (734/906) of HCWs had a low attitude score.

Practice scores ranged from 0% to 100%, with a mean of 57.76% ± 22.31%. 25% (226/906) of HCWs had a good practice score, and 24.20% (219/906) had a low practice score.

Table 2 presents statements related to HCWs’ knowledge, perceptions, attitudes, and practices regarding COVID-19 vaccines.

**Table 2:** Statements related to the HCWs’ knowledge and perception, attitudes, and practices regarding COVID-19 vaccines, Tunisia, 2024 (N = 906).

	Number	%
**HCWs’ knowledge and perception regarding COVID-19 vaccines**		
“Every year, COVID-19 can lead to hospitalizations, ICU admissions, and/or death in” *		
Healthcare workers	840	*92.72*
Persons with underlying chronic conditions	886	*97.79*
Individuals aged ≥ 65 years	887	*97.90*
“COVID-19 vaccines are safe for” *		
Healthcare workers	357	*39.40*
Persons with underlying chronic conditions	368	*40.62*
Individuals aged ≥ 65 years	374	*41.28*
“Getting the COVID-19 vaccine can reduce the chances of becoming severely ill in” *		
Healthcare workers	579	*63.91*
People with underlying chronic conditions	590	*65.12*
Individuals aged ≥ 65 years	592	*65.34*
“Can the COVID-19 vaccine protect against influenza infection?”		
Yes	137	*15.12*
No	769	*84.88*
**HCWs attitudes regarding COVID-19 vaccines**		
“The World Health Organization (WHO) currently recommends continued vaccination of frontline healthcare workers with COVID-19 vaccines. Will you continue to receive booster doses?”		
Yes	179	*19.76*
No	727	*80.24*
“If the COVID-19 vaccine becomes an annually recommended vaccine for health workers, like the seasonal influenza vaccine, will you be vaccinated every year with the COVID-19 vaccine?”		
Yes	175	*19.32*
No	731	*80.68*
“If COVID-19 becomes an annually recommended vaccine, will you recommend annual vaccination to your patients?”		
Yes	374	*41.28*
No	532	*58.72*
“If yes, who are the groups of people you would vaccinate or would recommend they receive the vaccine against COVID-19?”		
HCWs	66	*17.65*
Persons with an underlying chronic medical condition	324	*86.63*
Individuals aged > 65 years	313	*83.69*
“If the COVID-19 vaccine and seasonal influenza vaccine are offered together next influenza season (2024/2025) (co-administered), will you accept both?”		
I would accept the COVID-19 vaccine only	50	*5.52*
I would accept the Influenza vaccine only	205	*22.63*
I would not receive any vaccine	505	*55.74*
I would receive both	103	*11.37*
I do not have an opinion	43	*4.75*
**HCWs practices regarding COVID-19 vaccines**		
“Have you received a COVID-19 vaccine since the vaccines were introduced in your country?”		
Yes	874	*96.47*
No	32	*3.53*
“If your vaccine required more than one dose, did you complete your primary series/receive all your required doses?”		
Yes	764	*84.33*
No	142	*15.67*
“Did you receive one or more booster doses?”		
Yes	319	*35.21*
No	587	*64.79*
“COVID-19 booster/vaccine uptake during this current season 2023/2024?”		
Yes	38	*4.19*
No	867	*95.70*
“Did you recommend COVID-19 vaccines to your patients during the COVID-19 pandemic?”		
Yes	679	*74.94*
No	227	*25.06*
“Do you recommend a COVID-19 vaccination booster for your patients?”		
Yes	466	*51.43*
No	440	*48.57*

* Likert scale: “agree” and “strongly agree” were computed as “agree”. “strongly disagree”, “disagree”, and “do not have an opinion” were computed as “disagree”.

### 3.3. Univariable Analysis: Factors Associated with HCWs’ Good Attitudes and Practices Regarding COVID-19 Vaccines

In univariable analysis (Table 3), factors significantly associated with good knowledge and perception were urban areas, ≥13 years of professional experience, being a physician, and working in a department caring for high-risk patients. A positive attitude and good practices were also associated with strong knowledge and perceptions. HCWs from the central and southern parts of Tunisia had significantly lower odds ratios than those in the North. HCWs from district hospitals also had significantly lower odds ratios for good knowledge and perception than those in other types of healthcare settings (Table 3).

Factors significantly associated with a positive attitude were living in urban rather than rural areas, being ≥ 40 years old rather than younger, having ≥13 years of professional experience, being male, having at least one underlying non-communicable disease, or working in a department with high-risk patients. Having good knowledge and good practice were associated with a tenfold higher odds ratio of having a positive attitude.

Factors significantly associated with good practice were being older versus younger, having more years of professional experience, being male or a physician, having at least one underlying non-communicable disease, and working in a department with high-risk patients. Having good knowledge, perception, and attitude was associated with a tenfold increased odds ratio of good practice. HCWs from the central part of Tunisia and from the South showed significantly lower odds ratios of having good practice compared to those in the North. HCWs working in district hospitals had a significantly lower odds ratio of good practice compared to other types of healthcare settings.

### 3.4. Multivariable Analysis

**Figure 1:**
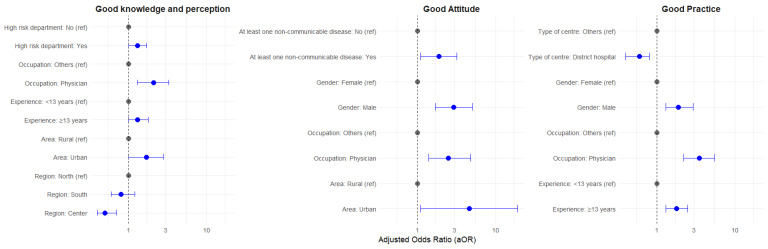
Factors associated with good knowledge and perception, positive attitude, and good practice of HCWs regarding COVID-19 vaccines in multivariable analysis, Tunisia 2024, N = 906.

Factors independently associated with good knowledge and perception were working in a rural areas compared to urban area (aOR = 1.73), more years of professional experience (aOR = 1.34), working as physician versus a nurse or other medical professional (aOR = 2.13), and working in departments with high-risk patients (aOR = 1.28) HCWs in the central region had a lower knowledge and perception compared to those in the North (Figure 1).

Factors independently associated with a positive attitude to COVID-19 vaccination were working in rural areas compared to urban areas (aOR = 4.58) (Figure 1), male HCWs compared to female (aOR = 2.97), physicians compared to nurses and other medical professionals (aOR = 2.56), and HCWs reporting at least one non-communicable disease compared to HCWs reporting having no non-communicable diseases.

Factors independently associated with good COVID-19 vaccination practice, as shown in Figure 1, were being male (aOR = 1.97), having more years of professional experience (aOR = 1.81), or working as a physician compared to nurses or other medical professionals (aOR = 3.49). HCWs from district hospitals had a lower odds ratio of good practice compared to the other types of PPHC.

## 4. Discussion

One third of HCWs in primary healthcare facilities in Tunisia had a good knowledge and perception score related to COVID-19 vaccines, while only 4.3% had a positive attitude score, and a quarter had a good practice score. Multivariable analysis identified several independent factors associated with these outcomes. Good knowledge and perception were independently associated with working in an urban area, having more years of professional experience, being a physician, and working in departments with high-risk patients. HCWs in the central region had lower knowledge and perceptions than those in the North. Factors associated with a positive attitude were working in an urban area, being a male HCW, having at least one underlying medical condition, and working as a physician. Further, good practice was independently associated with being a male HCW, having ≥13 years of professional experience, and working as a physician. HCWs in district hospitals had lower attitude scores compared to other types of hospitals.

One third of HCWs in our study had good knowledge and perception scores. This proportion is relatively low compared to findings from similar studies, such as a survey in southern Tunisia, where 65.3% of HCWs demonstrated good knowledge [15]. A national survey in Nepal found that 76% of HCWs had adequate knowledge about COVID-19 [21]. These differences may be attributed to variations in the definitions of good knowledge, geographical disparities, and the timing of the survey, as knowledge levels may have changed over time since the initial COVID-19 outbreak.

Greater professional experience (≥13 years) was independently associated with good knowledge and perception. This aligns with systemic reviews where professional experience significantly influenced COVID-19 knowledge levels and the expectation that senior staff accumulated more exposure to outbreak management and infection control practices [22].

In our study, only 4.3% had a positive attitude towards COVID-19 vaccines. This proportion is very low compared to other studies; a 2021 systematic review including 13 studies in different countries showed that COVID-19 vaccine acceptance ranged from 27.7% to 77.3% [23]. Another meta-analysis conducted in 2022 reported that pro-vaccination attitude towards COVID-19 vaccines among HCWs varied between 59.7% and 99.6% [24]. Although the percentages vary between populations, this could be explained by the timing of our survey in 2024, because after the end of the pandemic, HCWs might have been less willing to take COVID-19 vaccines compared to the pandemic period. Interestingly, our study showed that vaccine uptake declined among HCWs after the pandemic: 96.47% received a COVID-19 vaccine when it was introduced in the country, but only 35.21% received a booster dose. Less than 5% received a booster vaccine in the 2023–2024 season. This raises concerns for willingness to receive COVID-19 vaccines in future seasons. The reasons behind vaccine hesitancy should be explored in more depth in future studies. Information is currently sparse in Tunisia and other countries in the region, although one study reported that COVID-19 vaccine hesitancy was substantial among Africans and was based on perceived risk of coronavirus infection and past experiences, including knowing someone who had experience a series side effect from a vaccine in the past [25].

Factors that were associated with a positive attitude in our study were similar to those found in other studies; a systematic review including articles published between 2020 and 2022 found that factors such as male gender, advanced age, working as a physician, and having non-communicable diseases were associated with vaccine acceptance [26]. This could be explained by heightened personal risk perception, a factor that shows that self-perceived vulnerability was a key motivator for vaccine acceptance and thus, a better attitude [27].

A quarter of HCWs in our study had good practice scores regarding COVID-19 vaccines. This finding is considerably lower than a study conducted in southern Tunisia, which reported that 72.3% of HCWs had good COVID-19 vaccination practices [15]. This could be due to regional disparities in practices or differences in the surveys used in the studies. Additionally, timing and pandemic fatigue could have played a role. As our data were collected well after the end of the pandemic, HCWs might have grown complacent, leading to fewer meeting the criteria for “good practice” criteria. This could also be explained by the noted hesitancy to recommend COVID-19 vaccines for specific groups, such as children in Tunisia [28].

This study provided valuable national insight into the knowledge, attitudes, and practices of Tunisian HCWs regarding COVID-19. Random sampling and inclusion of all three main regions of Tunisia ensured representativeness. Furthermore, conducting the study in 2024 offers a timely reflection on post-pandemic vaccine attitudes and practices, especially in light of the emergence of new variants of concern. However, a limitation of our study was that it included only frontline HCWs from the public sector. Although COVID-19 vaccines were administered in the public sector, we acknowledge that private-sector HCWs also have an influential role in recommending vaccines. Although observational studies provide valuable descriptive results, causal inferences cannot be made, and there may be unmeasured or residual confounding, selection bias, and information bias. Conducting the survey face-to-face could have introduced social desirability bias in the responses, where participants might have provided answers they perceived as more socially acceptable. Another limitation of our study is the sampling method: we chose the most convenient PPHC when the expected number was not reached due to logistical constraints. This also influenced our sampling strategy by prioritizing HCWs from larger health districts while excluding those from smaller districts.

In light of these findings, we highlight the importance of strengthening HCWs’ knowledge through supportive policies and clear communication, while enabling good practice through adequate resources and leadership. Continuous education and training programs may be particularly beneficial for less experienced HCWs to improve their knowledge [29]. Targeting regions and areas with lower KAP metrics is important when implementing vaccination strategies and campaigns. Gaining HCWs’ trust in COVID-19 vaccines and combating misinformation are crucial to promoting good practices and, consequently, their ability to advise patients effectively [30]. This could improve not just KAP metrics but also healthcare outcomes, because improving one aspect of KAP can beneficially affect the others; for example, knowledge can boost attitude, which in turn drives good practices. Well-informed and motivated HCWs are more likely to adhere to infection control and accept vaccination, thereby enhancing the overall pandemic response and resilience of the health system. Finally, further studies that estimate the costs of immunization programs for HCWs are needed to better address vaccination strategies.

## 5. Conclusions

Our study found that only a minority of HCWs achieved good KAP scores, with 38% for knowledge and perception, 4% for attitude, and 25% for practice, indicating substantial gaps. Knowledge and perception were higher among HCWs in urban areas, those with more professional experience, physicians, and those working in departments with high-risk patients. We suggest targeted educational interventions in those groups and the need to extend interventions to urban and less experienced HCWs. The central region’s lower knowledge scores signal a need for region-specific interventions. Attitudes were generally poor, but better in rural areas and among male HCWs and those with underlying health conditions. However, low overall attitude scores call for initiatives to combat misinformation. Physicians with long professional experience tend to lead by example in safety practices, especially when they trust the vaccines and guidelines. Our findings underscore that investing in HCWs’ KAP is critical as it benefits the workers themselves and the communities they serve by ensuring safer practices and better preparedness for current and future public health challenges.

## Figures and Tables

**Table 1:** Sociodemographic characteristics of surveyed HCWs, Tunisia, 2024 (N = 906).

	Number	%
Governorate		
Beja	111	*12.25*
Gafsa	105	*11.59*
Kairouan	102	*11.26*
Kasserine	68	*7.51*
Kébili	104	*11.48*
Manouba	113	*12.47*
Monastir	112	*12.36*
Sidi-Bouzid	102	*11.26*
Tunis	89	*9.82*
Type of district		
Rural	63	*6.95*
Urban	843	*93.05*
Healthcare center type		
District hospitals	621	*68.54*
Other types	285	*31.46*
Gender		
Female	733	*80.91*
Male	173	*19.09*
Years of professional experience		
<13 years	400	*44.15*
≥13 years	506	*55.85*
Occupation		
Physicians	91	*10.04*
Nurse	636	*70.20*
Other	179	*19.76*
Department with high-risk patients		
Yes	527	*58.17*
No	556	*61.37*
HCWs with at least one self-reported non-communicable disease		
Yes	253	*27.92*
No	653	*72.08*

**Table 3:** Factors associated with good knowledge and perception, positive attitude, and good practice of HCWs regarding COVID-19 vaccines in univariable and multivariable analysis, Tunisia 2024, N = 906.

	Univariable Analysis			Multivariable Analysis		
Factors	Crude Odds Ratios (cOR)	95% CI	*p*-Value	Adjusted Odds Ratios (aOR)	95% CI	*p*-Value
**Good knowledge and perception**						
Region			<0.01			<0.01
North	1			1		
Center	0.45	0.33–0.61		0.50	0.36–0.69	
South	0.63	0.44–0.91		0.76	0.51–1.24	
Area			<0.01			0.04
Rural	1			1		
Urban	1.57	1.12–2.21		1.73	1.05–2.85	
Type of center			0.02	-	-	-
Others	1					
District hospital	0.82	0.69–0.97				
Age group (years)			0.19	-	-	-
<40 years	1					
≥40 years	1.12	0.94–1.33				
Gender			0.09	-	-	-
Female	1					
Male	1.19	0.98–1.44				
Experience (years)			0.02			0.05
<13 years	1			1		
≥13 years	1.21	1.02–1.44		1.34	1.01–1.78	
Occupation			<0.01			<0.01
Others	1			1		
Physician	1.60	1.31–1.96		2.13	1.26–3.28	
At least one non-communicable disease			0.94	-	-	-
No	1					
Yes	1.00	0.83–1.21				
High-risk department			0.01			0.05
No	1			1		
Yes	1.26	1.06–1.49		1.28	1.00–1.72	
Clinical or biological diagnosis of COVID-19 patients			0.06	-	-	-
Yes	1					
No	0.82	0.66–1.02				
Treatment of severely ill COVID-19 patients			0.13	-	-	-
Yes	1					
No	0.87	0.74–1.04				
Attitude			<0.01	-	-	-
Moderate or negative	1					
Positive	8.48	4.56–15.74				
Practice	1		<0.01	-	-	-
Moderate or low						
Good	5.44	3.93–7.54				
**Positive attitude**						
Region			0.09	-	-	-
North	1					
Center	0.74	0.44–1.26				
South	0.45	0.22–0.95				
Area			0.01			0.03
Rural	1			1		
Urban	4.94	1.19–20.44		4.58	1.09–19.19	
Type of center			0.4	-	-	-
Others	1					
District hospital	0.81	0.48–1.35				
Age group (years)			0.02	-	-	-
<40 years	1					
≥40 years	1.89	1.11–3.23				
Gender			<0.01			<0.01
Female	1			1		
Male	2.77	1.65–4.64		2.97	1.75–5.07	
Experience (years)			0.01	-	-	-
<13 years	1					
≥13 years	1.93	1.14–3.28				
Occupation			<0.01			<0.01
Others	1			1		
Physician	3.00	1.64–5.51		2.56	1.37–4.77	
At least one non-communicable disease			0.02			0.01
No	1			1		
Yes	1.81	1.09–2.99		1.92	1.14–3.23	
High-risk department			0.03	-	-	-
No	1					
Yes	1.74	1.04–2.91				
Clinical or biological diagnosis of COVID-19 patients			0.3	-	-	-
Yes	1					
No	0.74	0.39–1.37				
Treatment of severely ill COVID-19 patients			0.4	-	-	-
Yes	1					
No	0.81	0.49–1.34				
Knowledge and perception			<0.01	-	-	-
Moderate or low	1					
Good	8.48	4.56–15.74				
Practice			<0.01	-	-	-
Moderate or low	1					
Good	10.14	5.83–17.62				
**Good practice**						
Region			0.02	-	-	-
North	1					
Center	0.65	0.46–0.92				
South	0.65	0.43–0.97				
Area			0.24	-	-	-
Rural	1					
Urban	1.34	0.82–2.20				
Type of center			<0.01			<0.01
Others	1			1		
District hospital	0.56	0.41–0.77		0.58	0.42–0.82	
Age group (years)			<0.01	-	-	-
<40 years	1					
≥40 years	1.61	1.25–2.60				
Gender			<0.01			<0.01
Female	1			1		
Male	1.87	1.31–2.67		1.97	1.35–2.88	
Experience (years)			<0.01			<0.01
<13 years	1			1		
≥13 years	1.99	1.45–2.74		1.81	1.29–2.52	
Occupation			<0.01			<0.01
Others	1			1		
Physician	3.61	2.32–5.62		3.49	2.21–5.53	
At least one non-communicable disease			0.04	-	-	-
No	1					
Yes	1.40	1.01–1.94				
High-risk department			0.04	-	-	-
No	1					
Yes	1.37	1.01–1.86				
Clinical or biological diagnosis of COVID-19 patients			0.12	-	-	-
Yes	1					
No	0.75	0.51–1.08				
Treatment of severely ill COVID-19 patients			0.85	-	-	-
Yes	1					
No	0.97	0.71–1.32				
Knowledge and perception			<0.01	-	-	-
Moderate or low	1					
Good	5.44	3.93–7.54				
Attitude			<0.01	-	-	-
Moderate or negative	1					
Positive	10.14	5.84–17.92				

## Data Availability

Data is unavailable due to privacy or ethical restrictions.

## Supplementary Materials

The following supporting information can be downloaded at: https://www.mdpi.com/article/10.3390/vaccines14010074/s1, Tunisia KAP study.

## Abbreviations

The following abbreviations are used in this manuscript:

CAPI	Computer-Assisted Personal Interviewing
CDC	US Centers for Disease Control and Prevention
CI	Confidence Intervals
FETP	Field Epidemiology Training Program
HCW	Healthcare Workers
ICU	Intensive Care Units
IQR	Interquartile Ranges
ONMNE	National Observatory of New and Emerging Diseases
OR	Odd Ratios
PPHC	Primary Public Healthcare Centers
WHO	World Health Organization
