# Supplementary material for: COVID-19 Vaccination Knowledge, Attitudes, Perception, and Practices Among Frontline Healthcare Workers in Tunisia, 2024
Source: Vaccines (Basel). 2026 Jan 9;14(1):74. doi: 10.3390/vaccines14010074 (PMC12846571; doi:10.3390/vaccines14010074)
Supplement: Supplementary file 1 [file vaccines-14-00074-s001.zip › vaccines-4023376-supplementary.pdf]

Tunisia KAP study

Enquête sur les connaissances, attitudes, pratiques et perceptions (KAPP) des professionnels de santé sur la vaccination contre la COVID-19 (2023/2024), Tunisie

1. **Date de la visite du centre**

2. **Nom de l'enquêteur**

3. **Gouvernorat**

- ◯ Tunis
- ◯ Manouba
- ◯ Kasserine
- ◯ Béja
- ◯ Sidi-Bouzid
- ◯ Kairouan
- ◯ Gafsa
- ◯ Kébili
- ◯ Monastir

4. **Circonscription sanitaire**
5. **Delegation**
6. **Nom de l'établissement de santé**
7. **Type de milieu de l'établissement de santé ?**

- ◯ Rural
- ◯ Urbain

8. **Type d'établissement de santé**

- ◯ Centre type IV
- ◯ Centre Intermédiaire
- ◯ PMI
- ◯ Hôpital de circonscription

9. **La personne interrogée a-t-elle accepté l'entretien et signé le consentement ?**

- ◯ Oui
- ◯ Non

Section A : Caractéristiques sociodémographiques

[L'ENQUÊTEUR DIT À LA PERSONNE INTERROGÉE : Merci de m'avoir accordé votre temps. J'aimerais commencer par vous poser quelques questions sur vos caractéristiques sociodémographiques].

A1. Genre de l'enquêté

- ○ Homme
- ○ Femme

A2. Quel est votre âge ?

____

A3. Depuis combien d'années vous exercez dans le secteur de la santé en général ?

____

A4. Quelle est votre profession ?

- Médecin
- Infirmier
- Sage-femme
- Nutritionniste
- Aide-soignant
- Pharmacien
- Psychologue
- Autre

Autre, à préciser

____

A5. Dans quelle(s) spécialité, département(s) ou unité(s) travaillez-vous principalement ? (Sélectionnez tout ce qui s'applique)

- Médecine générale / interne
- Service des urgences
- Obstétrique / gynécologie
- Pédiatrie
- Radiologie
- Chirurgie
- ORL
- Stomatologie
- Autres

S'il vous plaît préciser

____

A6. Quelle catégorie de patients traitez-vous généralement ?

- Femmes enceintes
- Enfants < 18Ys
- Adultes souffrant de pathologies chroniques
- Adultes souffrant de pathologies infectieuses
- Personnes âgées (plus de 65 ans)
- Autres

veuillez préciser

____

A7a. Souffrez-vous d'une pathologie chronique ? Il peut s'agir, par exemple, d'obésité, de diabète, de maladies pulmonaires, d'hypertension artérielle ou d'une autre pathologie chronique.

- ○ Oui
- ○ Non
- ○ Je ne sais pas

A7b. De quelle(s) pathologies(s) chronique souffrez-vous ? (NE LISEZ PAS LES CHOIX DE RÉPONSES. COCHEZ TOUTES CELLES QUI S'APPLIQUENT)

- ☐ Obésité
- ☐ Diabète
- ☐ Maladie cardiovasculaire
- ☐ Maladie respiratoire
- ☐ Déficit immunitaire secondaire à une maladie ou une prise de médicaments immunosuppresseurs
- ☐ Hypertension artérielle
- ☐ Autre

Veuillez préciser

____

Section C : COVID19 et vaccination contre la COVID19

[L'INTERVIEWEUR : Merci d'avoir partagé vos réflexions sur la grippe et la vaccination contre la grippe. Je vais maintenant poser quelques questions sur la COVID-19 et la vaccination contre la COVID19.]

____

I. Connaissances Générales et Perceptions

____

C1. Avez-vous déjà confirmé le diagnostic (clinique ou biologique) de la COVID19 chez un patient ?

- ○ Oui
- ○ Non
- ○ Je ne me souviens pas

C2. Avez-vous déjà traité un patient ayant développé une complication grave (pneumonie ou décès) nécessitant une hospitalisation en rapport avec la COVID19 ou une suspicion de COVID19 ?

- ○ Oui
- ○ Non
- ○ Je ne me souviens pas

Ensuite, il y a une série d'affirmations sur la gravité du COVID19, la sureté et l'efficacité des vaccins contre la COVID19 pour plusieurs groupes de personnes. Veuillez indiquer dans quelle mesure vous êtes d'accord ou en désaccord avec chaque affirmation pour les différents groupes de personnes.

____

**C3. "Chaque année, la COVID19 peut entraîner des hospitalisations, des admissions en unité de soins intensifs et/ou des décès pour ces groupes ci-dessous :**	Tout à fait d'accord	d'accord	Être en désaccord	Pas du tout d'accord	Sans opinion
**Personnels de santé**	○	○	○	○	○

**Population générale**	◯	◯	◯	◯	◯
**Femmes enceintes**	◯	◯	◯	◯	◯
**Le fœtus d'une femme enceinte**	◯	◯	◯	◯	◯
**Personnes atteintes de maladies chroniques**	◯	◯	◯	◯	◯
**Personnes âgées (65 ans et plus)**	◯	◯	◯	◯	◯
**Enfants d'âge scolaire**	◯	◯	◯	◯	◯
**Moi**	◯	◯	◯	◯	◯
**Enfants <5 ans**	◯	◯	◯	◯	◯
**Les personnes avec un déficience immunitaire secondaire à une maladie ou à une prise médicamenteuse**	◯	◯	◯	◯	◯

**C4. Veuillez indiquer à quel point vous pensez que le vaccin contre la COVID19 est sûr pour chacun des groupes ci-dessous.**	Très sûr	Sûr	Dangereux	Très dangereux	Sans opinion
**Personnels de santé**	◯	◯	◯	◯	◯
**Population générale**	◯	◯	◯	◯	◯
**Femmes enceintes**	◯	◯	◯	◯	◯
**Personnes atteintes de maladies chroniques**	◯	◯	◯	◯	◯
**Personnes âgées (65 ans et plus)**	◯	◯	◯	◯	◯
**Enfants <5 ans**	◯	◯	◯	◯	◯
**Enfants d'âge scolaire**	◯	◯	◯	◯	◯
**Moi**	◯	◯	◯	◯	◯
**le foetus d'une femme enceinte**	◯	◯	◯	◯	◯
**Les personnes avec un déficience immunitaire secondaire à une maladie ou à une prise médicamenteuse**	◯	◯	◯	◯	◯

**C5. "Le fait de recevoir le vaccin contre la COVID19 peut réduire le risque des formes graves (c'est-à-dire nécessitant une hospitalisation) pour chacun des groupes énumérés ci-dessous :**	Tout à fait d'accord	D'accord	Être en désaccord	Pas du tout d'accord	Sans Opinion
**Personnels de santé**	◯	◯	◯	◯	◯
**Population générale**	◯	◯	◯	◯	◯
**Femmes enceintes**	◯	◯	◯	◯	◯
**Personnes atteintes de maladies chroniques**	◯	◯	◯	◯	◯
**Personnes âgées (65 ans et plus)**	◯	◯	◯	◯	◯

**Enfants <5 ans**	○	○	○	○	○
**Enfants d'âge scolaire**	○	○	○	○	○
**Moi**	○	○	○	○	○
**le foetus d'une femme enceinte**	○	○	○	○	○
**Les personnes avec un déficience immunitaire secondaire à une maladie ou à une prise médicamenteuse**	○	○	○	○	○

II. Acceptation de la vaccination contre la COVID19 par le personnel de santé

C6. Avez-vous déjà reçu un vaccin contre la COVID19 depuis qu'il a été introduit en Tunisie ?

- ○ Oui
- ○ Non
- ○ Je ne me souviens pas

C.6.1.Pourquoi?

____

C7. Si le vaccin nécessitait plus qu'une dose, avez-vous terminé votre série primaire/reçu toutes les doses primaires requises ?

- ○ Oui
- ○ Non
- ○ Je ne me souviens pas

C8. Pourquoi vous n'avez pas terminé la série de vaccinations ?

____

C9. Avez-vous reçu une ou plusieurs doses de rappel ?

- ○ Oui
- ○ Non
- ○ Je ne me souviens pas

C9. 1. Combien de doses de rappel vous avez reçu ?

____

C9. 2. Pourquoi vous n'avez pas reçu les doses de rappel ?

____

C10. L'Organisation mondiale de la santé (OMS) recommande actuellement de poursuivre la vaccination contre la COVID19 chez les personnels de santé de première ligne. Continuerez-vous à recevoir des doses de rappel ?

- Oui
- Non
- Je n'ai pas d'opinion particulière

C11. Si le vaccin contre la COVID-19 devient un vaccin recommandé chaque année pour le personnel de santé, au même titre que le vaccin contre la grippe saisonnière, feriez-vous le vaccin chaque année avec le vaccin COVID-19 ?

- Oui
- Non
- Je n'ai pas d'opinion particulière

C12. Qu'est-ce qui vous a encouragé à recevoir le vaccin contre la COVID19 ?

- Il était offert gratuitement dans mon travail (pas obligatoire)
- Il était obligatoire dans mon travail
- Pour protéger mes patients
- Pour me protéger de l'infection
- Pour protéger ma famille
- C'est recommandé par le ministère de la santé ou mon organisation
- Autre

Veuillez préciser

____

C13. Qu'est-ce qui vous a découragé à recevoir le vaccin contre la COVID19 ?

- Le vaccin n'est pas disponible pour le personnel de santé dans mon pays
- Je ne pense pas qu'il me protégera contre l'infection
- Je ne voulais pas payer pour cela (non gratuit au travail)
- Je n'avais pas le temps d'aller me faire vacciner
- Je ne pense pas être susceptible à la COVID19
- Je ne crois pas à l'efficacité des vaccins
- Je ne pense pas que le vaccin contre la COVID19 soit sûr
- Ce n'est pas recommandé par le ministère de la santé ou mon organisation
- Je préfère l'immunité naturelle
- Autre

Veuillez préciser

____

C14. Recevez-vous le vaccin contre la COVID-19/dose de rappel durant cette saison 2023/2024

- Oui
- Non

III. Recommandation des Vaccins contre la COVID19 par les professionnels de la Santé

C15. Avez-vous recommandé la vaccination contre la COVID-19 à vos patients pendant la pandémie COVID-19 ?

- Oui
- Non
- Ne souhaite pas répondre

C15.a. Si non, Pourquoi n'avez-vous pas recommandé la vaccination contre la COVID-19 à vos patients ?

C16. Recommandez-vous à vos patients un rappel de la vaccination contre la COVID-19 ?

- Oui
- Non

C16.A. Si non, Pourquoi ?

C17. S'il était recommandé de recevoir le vaccin contre la COVID19 chaque année, recommanderiez-vous à vos patients de recevoir le vaccin ?

- Oui
- Non
- Je n'ai pas d'opinion spécifique

C18. Quels sont les trois groupes de personnes chez qui vous recommandez le vaccin contre la COVID19 [L'INTERVIEWEUR : NE PAS LIRE LA LISTE À VOIX HAUTE].

- ☐ Les adultes en bonne santé
- ☐ Les personnes âgées (65 ans et plus)
- ☐ Les enfants d'âge scolaire (5-16 ans)
- ☐ Les enfants de moins de 5 ans
- ☐ Les femmes en âge de procréer
- ☐ Les femmes enceintes
- ☐ Les travailleurs de la santé
- ☐ Les personnes atteintes du VIH/SIDA
- ☐ Les personnes atteintes de la tuberculose
- ☐ Les personnes avec un déficience immunitaire secondaire à une maladie ou à une prise médicamenteuse
- ☐ Les personnes atteintes de pathologies chroniques
- ☐ Je ne sais pas
- ☐ Autres

Autres, à préciser

____

C19 Quelle est la raison la plus importante qui vous pousse à recommander le vaccin contre la COVID19 ?

____

C20. Quelle est la raison la plus importante qui vous pousse à ne pas recommander le vaccin contre la COVID19?

____

C21. Avez-vous rencontré une résistance ou une hésitation de la part des patients lors de la recommandation du vaccin contre la COVID19 ?

- ○ Oui
- ○ Non
- ○ Je ne me souviens pas

C21.1 Quelle(s) sont les raison(s) données par les patients concernant leur résistance ou l'hésitation à recevoir le vaccin contre la COVID19 ? (Cochez toutes les réponses qui s'appliquent)

- ☐ Le vaccin n'est pas gratuit
- ☐ Le centre de vaccination est loin de chez moi
- ☐ Les heures de travail du centre de vaccination ne me conviennent pas
- ☐ Temps d'attente prolongé au centre de vaccination
- ☐ Histoire d'événements indésirables liés aux vaccins contre la COVID19 chez la personne
- ☐ Histoire d'événements indésirables liés aux vaccins contre la COVID19 chez un autre membre de la famille
- ☐ Le vaccin n'est pas sûr
- ☐ Le vaccin n'est pas efficace
- ☐ Autre

Autre, à préciser

____

C22. Est-ce que le vaccin contre la COVID-19 protège de la grippe ?

- ○ Oui
- ○ Non
- ○ Je ne sais pas

Section D : Expérience avec le vaccin contre la grippe

[INTERVIEWER : Merci de nous avoir fait part de votre opinion sur la COVID-19. Je vais maintenant vous poser quelques questions spécifiquement liées à votre expérience de la vaccination contre la grippe e].

D3. Si le vaccin contre la COVID-19 et le vaccin contre la grippe sont offerts ensemble lors de la prochaine saison grippale (2024/2025) (co-administration), accepteriez-vous les deux ?

- Oui, je recevrais les deux
- Non, je ne recevrais ni l'un ni l'autre
- Non, je n'accepterais que le vaccin contre la grippe
- Non, je n'accepterais que le vaccin contre la COVID-19
- Je n'ai pas d'opinion particulière

Veuillez expliquer pourquoi

Section E : Commentaires

E1. Avez-vous des commentaires ou des suggestions concernant le vaccin contre la grippe pour les professionnels de la santé ?

E2. Avez-vous des commentaires ou des suggestions concernant le vaccin contre la COVID-19 pour les professionnels de la santé ?

Je vous remercie pour votre participation à l'enquête

Merci pour votre temps
